# Efficacy of intrathecal methotrexate in children with high-risk medulloblastoma over three years: a retrospective study from a single center

**DOI:** 10.1007/s11060-023-04388-2

**Published:** 2023-07-20

**Authors:** Yu-Tong Zhang, Yu Wang, Xiao-dan Zhong, Jian Chang

**Affiliations:** Department of Pediatric Oncology, the First Hospital of Jilin University, Changchun, Jilin 130021 China

**Keywords:** Medulloblastoma, High risk, Intrathecal methotrexate, Leukoencephalopathy

## Abstract

**Purpose:**

Chemotherapy is commonly used for treatment in children over three years old with high-risk medulloblastoma(MB). However, little is currently known about the therapeutic benefits and side effects of intrathecal methotrexate(MTX), warranting further research.

**Methods:**

In this retrospective study, patients who received intrathecal MTX during chemotherapy were included in the MTX group (n = 32), and patients that only underwent cerebrospinal fluid (CSF) cytology analysis were assigned to the control group (n = 14).

**Results:**

In the MTX group, 27(84.38%) patients had metastatic disease, 3(9.38%) had diffuse anaplasia, and 3(9.38%) had residual disease greater than 1.5 cm^2^. Molecular subgroup classification was available for 28(87.5%) patients. In the control group, 8(57.14%) patients had metastatic disease, 3(27.27%) had diffuse anaplasia, and 6(42.86%) had residual disease greater than 1.5 cm^2^. Molecular subgroup classification was available for 6(42.86%) patients. The 5-year progression-free survival was 70.99% and the 5-year overall survival was 72.99% for the MTX group, and the corresponding values were 41.67% and 50% for the control group, respectively. 6 (18.75%) patients in the MTX group with group 4 disease developed MTX-related acute leukoencephalopathy and one of them died.

**Conclusions:**

Our findings support the addition of intrathecal MTX during chemotherapy as the optimal management for children with group 3 and SHH high-risk MB. However, it is not recommended for group 4 MB patients, especially in resource-limited regions.

**Trial registration number:**

: Retrospective registered No.(2020 − 117).

**Supplementary Information:**

The online version contains supplementary material available at 10.1007/s11060-023-04388-2.

## Introduction

Medulloblastoma (MB) is widely acknowledged as one of the most common malignant brain tumors in children [1]. Approximately one-third of children are diagnosed with high-risk MB, associated with poor survival, especially in resource-limited regions. It has been reported that the 5-year progression-free survival (PFS) rate is above 60% with comprehensive treatment in developed countries [2]. In contrast to standard-risk MB, no consensus has been reached on the optimal treatment for high-risk MB, although recent evidence suggests that treatment based on high-dose chemotherapy and stem cell transplantation combined with conventional radiotherapy can result in a high survival rate in children with newly diagnosed high-risk MB [3, 4].

However, autologous stem-cell transplantation is not routinely performed in our institution due to its high cost and associated risks. In this regard, death has been reported in 6–14% of patients that undergo autologous stem-cell transplantation [5]. Accordingly, methotrexate (MTX) intrathecal injections have been adopted for high-risk MB. Due to the relative lack of data concerning the efficacy and toxicity of MTX intrathecal injections in central nervous system (CNS) embryonal tumors, it remains controversial whether MTX intracranial injection should be administered. MTX has been used in European trials but is rarely adopted in the United States because of its association with radiation-induced leukoencephalopathy. To our knowledge, few study has hitherto reported acute leukoencephalopathy induced by MTX treatment for MB during chemotherapy [6].

Herein, a retrospective study was conducted to evaluate the therapeutic benefits of intrathecal MTX in children aged over three years with high-risk MB to objectively assess the intervention and side effects [7], especially leukoencephalopathy. We anticipate that this approach may improve or maintain the survival outcomes of patients with high-risk MB over 3 years of age, similar to those of developed countries, given that stem cell transplantation is unavailable in resource-limited regions.

## Materials and methods

### Patients

This study was approved by the Ethics Committee of our hospital. We retrospectively reviewed the medical records of patients over 3 years diagnosed with high-risk MB at our hospital between January 1st, 2010 and December 31st, 2018. High-risk MB was defined based on the following criteria: residual disease > 1.5 cm^2^, diffuse anaplasia histology, and metastatic spread of disease (M0, 1, 2, 3) according to Chang’s classification [8]. The patient population was divided into two groups: the MTX group, consisting of patients who received intrathecal MTX, and the control group, consisting of those who did not. Patients in the WNT subgroup were excluded due to prior favorable treatment responses.

The following data were extracted from each patient, including age, gender, histological subtype, tumor stage, risk group, molecular subgroup, chemotherapy regimen, and dosage, details of MTX intracranial injection, clinical manifestation of leukoencephalopathy, treatment regimen of leukoencephalopathy, and patient outcomes (including the cause of death). Results for periodic MRI scanning, comprising a minimum of T1, T2 and T1 with gadolinium sequences were documented for evaluation of leukoencephalopathy during and after treatment [6]. Leukoencephalopathy was defined as the neuroimaging leukoencephalopathy toxicity grading criteria [9]. Data collection from the clinical records of patients was approved by the Institutional Review Board. All data were anonymous, and the need for informed consent was waived due to the retrospective observational nature of this study. Nonetheless, written informed consent was obtained from the parents or legal guardians of patients before the initiation of chemotherapy.

### Statistical analysis

PFS and overall survival (OS) were estimated by the Kaplan-Meier method with Rothman’s 95% confidence interval (CI). Meanwhile, the median follow-up was estimated by the reverse Kaplan-Meier method. Hazard ratios (HR) and 95% CIs of multivariate Cox proportional hazard models were used to explore the effects of MTX intrathecal injection. Nonparametric data were compared by the Mann-Whitney U test. The statistical significance level was set at *p* < 0.05. GraphPad Prism 9.4 was employed for all statistical and image analyses.

## Results

### Patients

From January 1st, 2010 to December 31st, 2018, 46 patients aged over 3 years were diagnosed with high-risk MB with male predominance (n = 33, 71.7%) and a median age at diagnosis of 10.6 (range, 5–21) years. Of the 46 MB patients, 34 individuals (73.9%) were included in the molecular analyses for further biological investigations. If the parents agreed to receive intrathecal methotrexate, then the patients would receive MTX. Otherwise, intrathecal methotrexate was not performed. As such, 32 patients received intrathecal MTX and were classified in the MTX group, while the remaining 14 were treated without intrathecal MTX and were assigned to the control group.

Among patients in the MTX group, 27 (84.38%) had metastatic disease, 3 (9.38%) had diffuse anaplasia [10], and 3 (9.38%) had residual disease greater than 1.5 cm^2^. Molecular subgroup classification was available in 28 (87.5%) patients, which assigned patients to the following subgroups: WNT (n = 0), SHH (n = 5), group 3 (n = 6), and group 4 (n = 17).

Among the 14 patients in the control group, 8 (57.14%) had metastatic disease, 3 (27.27%) had diffuse anaplasia, and 6 (42.86%) had residual disease greater than 1.5 cm^2^. Molecular subgroup classification was available in 6 (42.86%) patients, assigning patients to the following subgroups: WNT (n = 0), SHH (n = 2), group 3 (n = 1), and group 4 (n = 3).

More details on these patients are presented in Supplementary Table 1.

### Treatments

All patients initially underwent maximum safe resection of the tumor, followed by craniospinal radiotherapy at a dose of 36 Gy with a boost to the posterior fossa, reaching a cumulative dose of 55.8 Gy with conventional fractionation at 1.8 Gy/d. Radiotherapy was initiated within 31 days after diagnostic surgery for all patients.

**Fig. 1:**
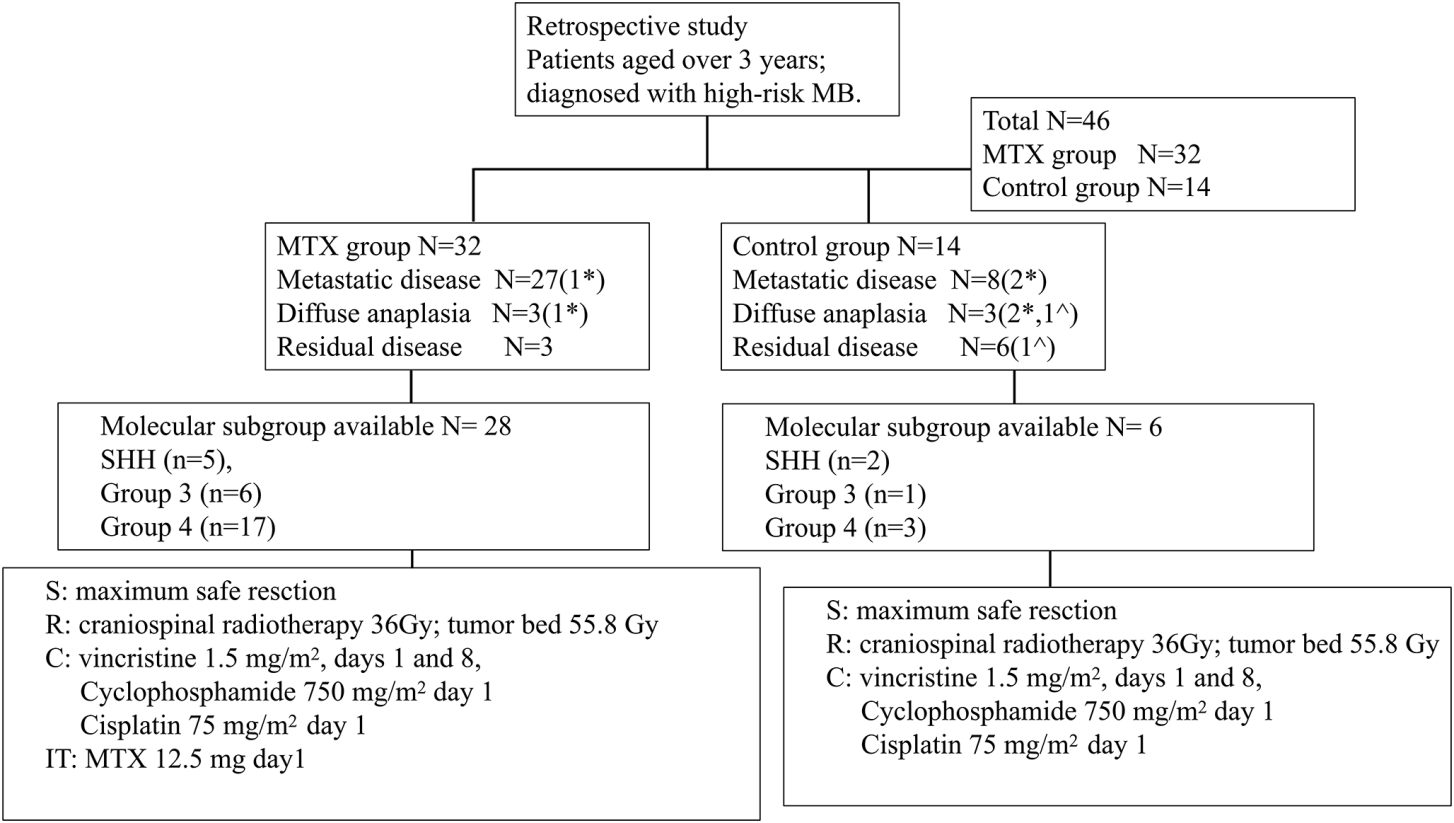
Consort diagram of this study. * One patient betlongs to both groups. ^ One patient betlongs to both groups. S, surgery. R, radiation, C, chemotherapy

9 patients in the MTX group refused to undergo chemotherapy after the completion of radiotherapy based on their parents’ decision. Unfortunately, these patients relapsed 6 to 12 months after radiotherapy and underwent a second surgery, followed by chemotherapy within 4 to 6 weeks after surgery, following the same procedure as other patients in the study. The remaining 34 patients underwent 8 cycles of chemotherapy after a 4-6-week rest while completing radiotherapy. The chemotherapy regimen consisted of intravenous administration of 1.5 mg/m^2^ vincristine over 15 min on days 1 and 8, 750 mg/m^2^ cyclophosphamide over 3 h on days 1 and 2, and 75 mg/m^2^ cisplatin on day 1. A single dose of MTX at 12.5 mg (2.5 mg per milliliter of 0.9% sodium chloride solution) was administered through lumbar puncture on day 1 in the MTX group, while the control group underwent cerebrospinal fluid (CSF) cytology analysis alone. Brain magnetic resonance imaging (MRI) examination and spine MRI were performed routinely after two, four, six, and eight cycles of chemotherapy for all patients. Consort diagram of this study is presented in Fig. 1.

### Outcomes

The 5-year PFS was 70.99% (95% CI, 58.22-90.42%), and the 5-year OS was 72.99% (95% CI, 60.41-93.06%) for the MTX group, while the corresponding values for the control group were 41.67% (95% CI, 17.93-66.92%) and 50% (95% CI, 27.88-77.14%), respectively. A Chi-square test revealed a significant difference in the response rate between the two groups when comparing PFS and OS (*p* < 0.05).

Factors associated with OS were analyzed. It was found that superior OS was observed in children with R0 resection (HR: 0.48, 95% CI: 0.0053–33.29), M0 status (HR: 0.59, 95% CI: 0.00092-28.87), no diffuse anaplasia histology (HR: 0.14, 95% CI: 0.0058-1.53) and MTX intrathecal injection (HR: 0.49, 95% CI: 0.099–2.49).

Besides, a superior PFS was noted among children with R0 resection (HR: 0.46, 95% CI: 0.0052–31.26), had M0 status (HR: 0.44, 95% CI: 0.0069–21.49), no diffuse anaplasia histology (HR: 0.14, 95% CI: 0.0060–1.52) and received MTX intrathecal injection (HR: 0.67, 95% CI: 0.141–3.39).

However, none of these factors were identified as significant risk factors for OS or PFS (*p* > 0.05).

#### MTX-related acute leukoencephalopathy

Six (18.75%) of the 32 patients developed MTX-related acute leukoencephalopathy. More details on these six patients are presented in Table 1. Overall, the symptoms of acute leukoencephalopathy were relatively mild with only one patient (Case 1) presenting with alalia, two (Cases 3, 4) were asymptomatic and three (Cases 2, 5, 6) exhibited fatigue, which was initially overlooked until the diagnosis of leukoencephalopathy. Five patients (Cases 2, 3, 4, 5, and 6) were diagnosed with leukoencephalopathy during routine MRI scans after undergoing 6, 4, 4, 4, and 6 cycles of chemotherapy, respectively.

**Table 1:** The details of the six patients developed MTX-related acute leukoencephalopathy

												Radiation(Gy)															
No.	Age/ Gender	Location of primary tumor		tumor size(cm)		Extend of resection		M status	Histology			CSI	Primary tumor	molecular subgroup			presentation		time of leu		diagnose of leu		treatment of leu		Outcome(years)		
1(3)	9.5/F	fourth ventricle		5.4 × 4.6		R+		M0	classic			36	54	Group 4			alalia		after cycle 3		MRI		IVIG + methyl prednisolone		OS(4.4)		
2(7)	8.8/F	cerebellar hemisphere		3.2 × 4.1		R0		M1	classic			36	54	Group 4			fatigue		after cycle 6		MRI + ASL		IVIG + methyl prednisolone		DOD(0.5)		
3(8)	10.3/F	cerebellar hemisphere		3.5 × 4.0		R0		M1	classic			36	54	Group 4			asymptomatic		after cycle 4		MRI + ASL		IVIG + methyl prednisolone		OS(4.7)/P		
4(13)	8.7/F	fourth ventricle		2.9 × 3.0		R0		M1	classic			36	54	Group 4			asymptomatic		after cycle 4		MRI + ASL		IVIG + methyl prednisolone		OS(4.8)		
5(16)	8.3/F	fourth ventricle		3.1 × 5.2		R0		M1	classic			36	54	Group 4			fagitue		after cycle 4		MRI + ASL		IVIG + methyl prednisolone		OS(4.8)		
6(17)	7.8/F	fourth ventricle		2.3 × 3.2		R0		M1	classic			36	54	Group 4			fagitue		after cycle 6		MRI + ASL		IVIG + methyl prednisolone		OS(3.9)		

**Fig. 2:**
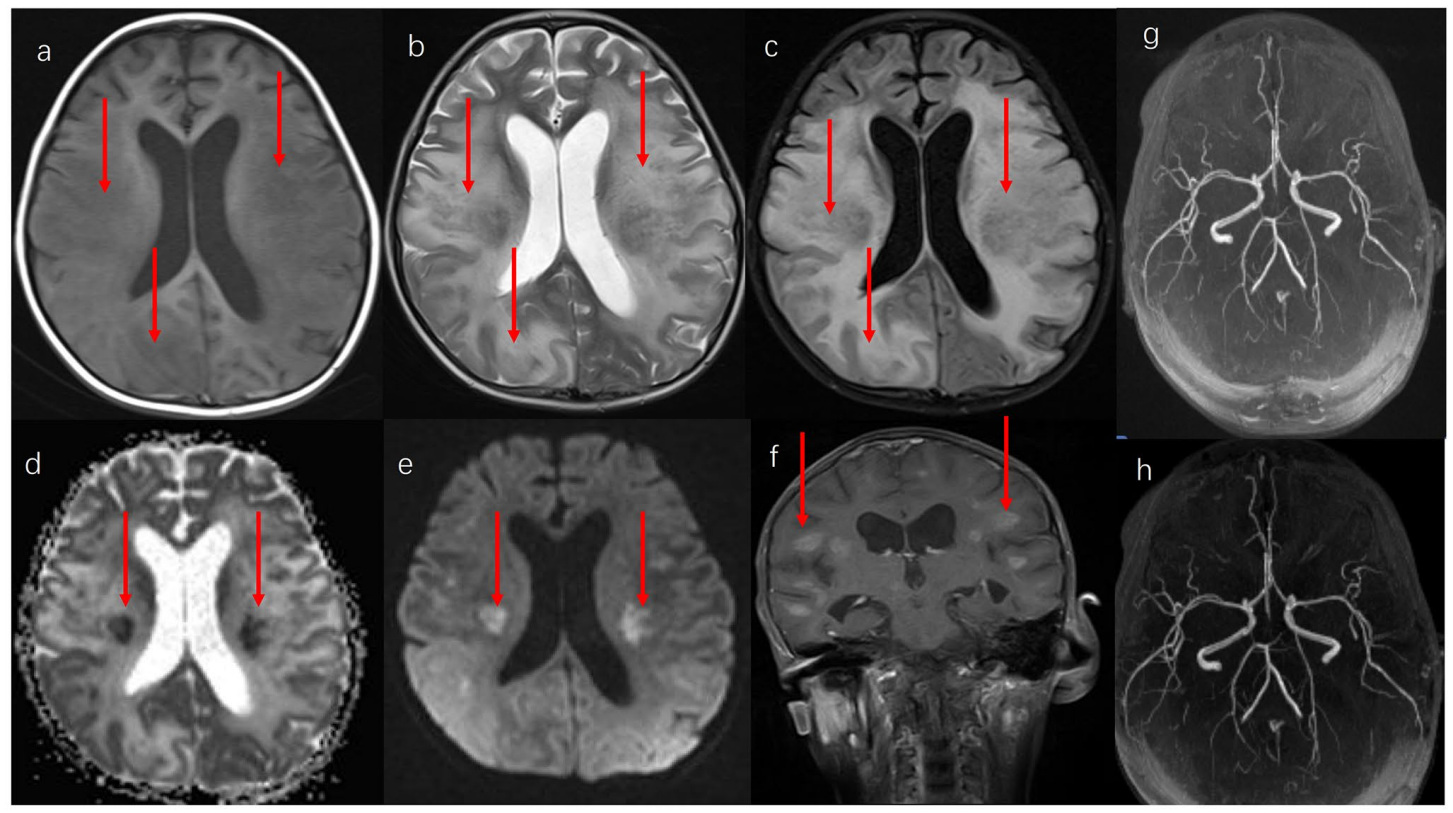
Brain MRI revealed multiple lesions in the bilateral cerebellar hemispheres, thalamus, basal ganglia region, lateral ventricle, radial coronal, centrum semiovale, bilateral frontoparietal temporal lobe, and right occipital lobe. a: T1 weighted imaging (T1W1) revealed slightly low signal. b: T2 weighted imaging (T2WI) showed high signal. c: Flair image presented a slightly high signal intensity with blurred boundaries. d and e: Diffusion-weighted imaging (DWI) suggested limited diffusion of some lesions. f: Enhanced MTI suggested mild enhancement of some white matter lesions. g and h: There was no obvious abnormality on the brain vessel MRI

In Case 1, tumor cells were documented in the CSF after one cycle of chemotherapy. MTX (12.5 mg) was injected intravenously once a week for antitumor treatment. Three single doses were administered to this patient during the interval between two cycles of chemotherapy. However, the patient developed alalia shortly after and could not proceed with the next cycle of chemotherapy. Brain MRI revealed multiple lesions in the bilateral cerebellar hemispheres, thalamus, basal ganglia region, lateral ventricle, radial coronal, centrum semiovale, bilateral frontoparietal temporal lobe, and right occipital lobe. T1 weighted imaging (T1W1) revealed a slightly low signal intensity (Fig. 2a), T2 weighted imaging (T2WI) showed high signal intensity (Fig. 2b), while the Fluid-attenuated inversion recovery (FLAIR) image presented a slightly high signal with blurred boundary (Fig. 2c). Diffusion-weighted imaging (DWI) suggested restricted diffusion in some lesions (Fig. 2d, e), and enhanced MTI suggested mild enhancement of some white matter lesions (Fig. 2f). The brain vessel MRI showed no significant abnormality (Fig. 2g, h). Oligoclonal band (OCB) analysis found the presence of immunoglobulin G (IgGs) in the patient’s CSF, leading to a diagnosis of leukoencephalopathy was diagnosed. Accordingly, she was treated with intravenous methylprednisolone at 2 mg/kg three times daily (tapered over 2 weeks), oxygen support, and human blood albumin injection. At the latest follow-up, the patient’s condition remained stable.

**Fig. 3:**
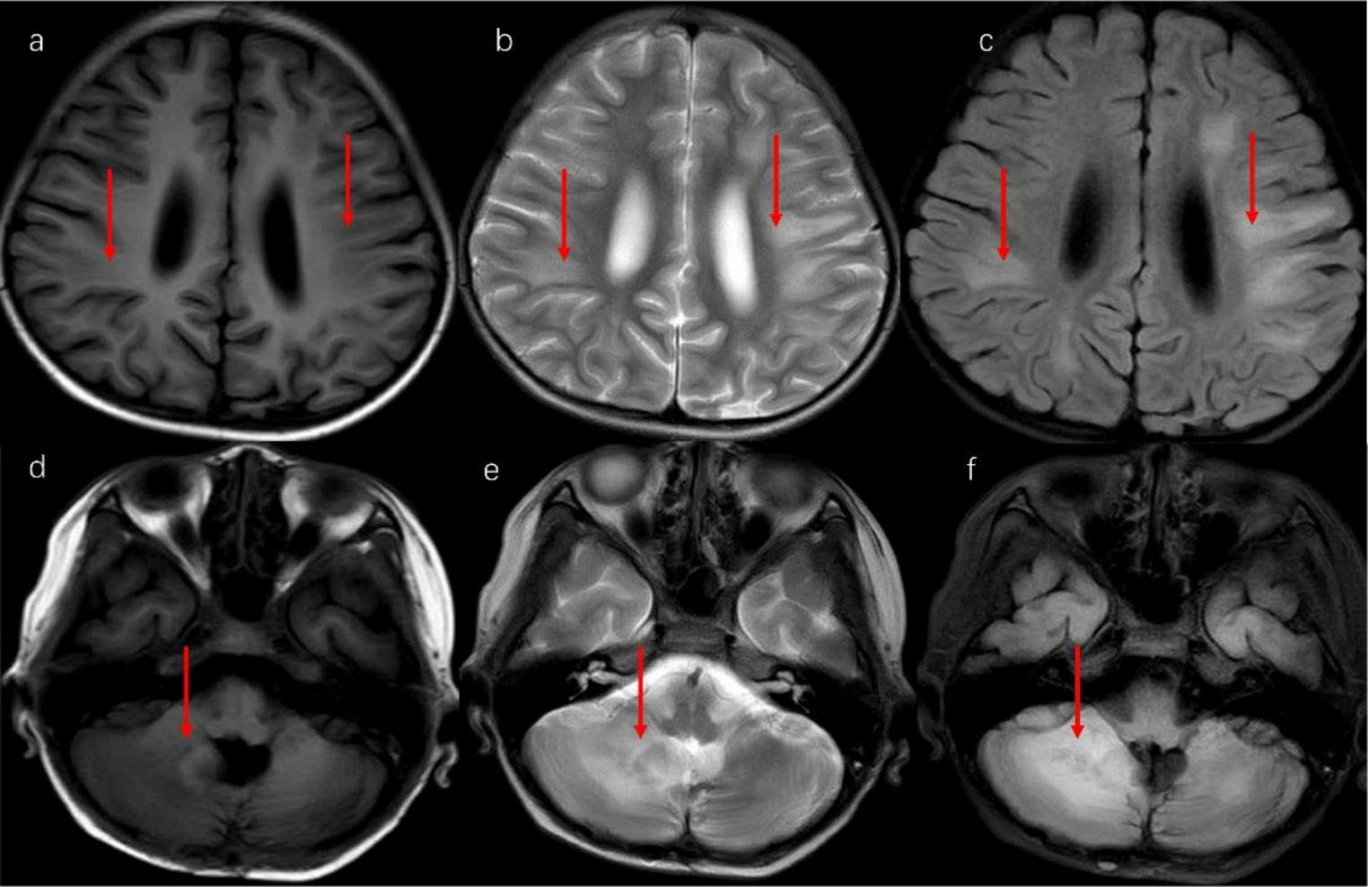
Several infiltrative lesions involved the bilateral frontal, parietal, temporal lobes, right cerebellar hemisphere, and left pontile of Case 2. a and d: T1WI showed a slightly low signal intensity. b and e: T2WI displayed a high signal intensity. c and f: Flair image revealed a slightly high signal intensity

**Fig. 4:**
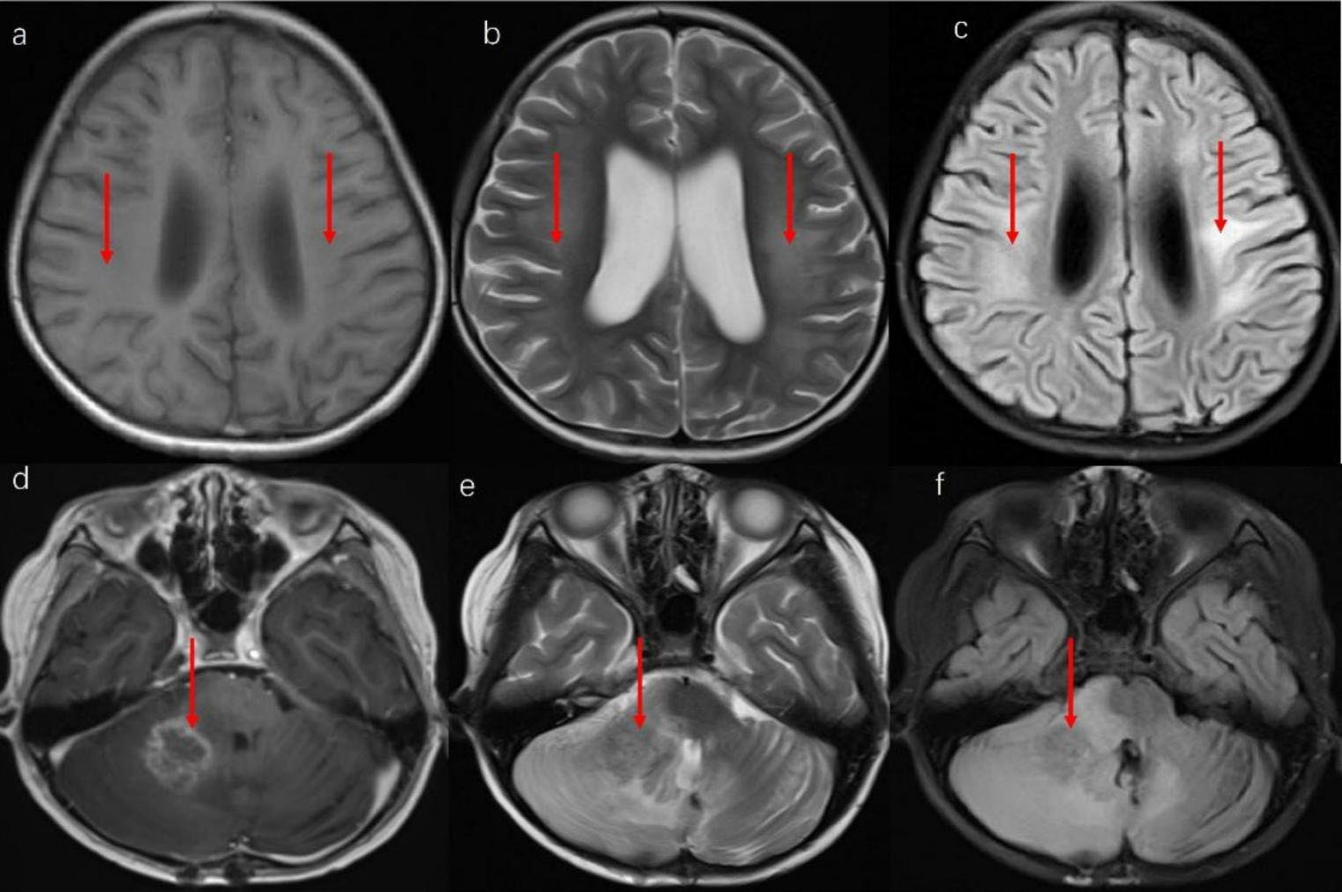
Several infiltrative lesions involved the bilateral frontal, parietal, temporal lobes, right cerebellar hemisphere, and left pontile of Case 2 (1 month after treatment). a.b,c: the abnormal signal in the white matter area was slightly improved compared with Fig. 2. d,e,f: the cerebellar hemisphere showed a 2.5 cm enhance with contrast enhancement

In Case 2, a screening MRI conducted after the 6th cycle of chemotherapy revealed several infiltrative lesions that involved the bilateral frontal, parietal, temporal lobes, right cerebellar hemisphere, and left pontile. T1WI showed a slightly low signal intensity (Fig. 3a, d), T2WI displayed a high signal intensity (Fig. 3b, e), while the flair image revealed a slightly high signal intensity (Fig. 3c, f). The largest size of the lesion was about 1.5 cm, located in the right cerebellar hemisphere. Magnetic resonance spectroscopy (MRS) revealed a Cho/NAA ratio of 1.78 and 0.97 in the lesion of the right cerebellar hemisphere and the left lateral ventricle, respectively, which raised the possibility of tumefactive demyelinating lesions and recurrence of medulloblastoma. Cerebral blood flow (CBF) was measured, but no abnormal signal was observed during CBF subsequently. Accordingly, a diagnosis of leukoencephalopathy was established. The patient received dexamethasone at a dose of 10 mg/d and oxygen support at a local hospital. After a month, the abnormal signal in the white matter area was slightly improved (Fig. 4a, b, c). However, the lesions in the cerebellar hemisphere increased to 2.5 cm with contrast enhancement (Fig. 4d, e, f). MRS revealed that the Cho + Cr/NAA ratio of this lesion was 5.3. Besides, CBF revealed multiple cerebral hyperperfusion lesions, and relapsed disease was suspected. Unfortunately, the patient died two weeks later.

##### Cases

3, 4, 5, and 6 showed similar findings to Case 1, with all patients being alive but presenting with severe neurological symptoms.

None of the patients in the control group developed acute leukoencephalopathy and all of them complete all the the proposed treatment. Except for acute leukoencephalopathy, there was no difference in the prevalence of high-grade toxicity (grade ≥ 3) between the two groups.

## Discussion

MTX has been associated with clinical or neuroradiological evidence of leukoencephalopathy in survivors of childhood acute lymphoblastic leukemia (ALL) and CNS or head and neck tumors. Current evidence suggests an impaired low intelligence quotient (IQ) is strongly related to the reduced white matter volume in MB patients treated with chemotherapy and radiotherapy after surgery [11]. A recent study documented the long-term neurocognitive outcomes of young children with ALL receiving CNS-directed therapy consisting of chemoradiotherapy, high-dose intravenous MTX, and very high-dose intravenous MTX in addition to intrathecal therapy. Although multiple drugs have been applied in addition to MTX, the acute neurotoxicity reported in cancer patients undergoing therapy is usually attributed to MTX. However, a clinical trial showed intraventricular methotrexate therapy was feasible and mostly well tolerated as part of primary therapy for children with infant and/or metastatic MB. A higher cumulative dose of intraventricular methotrexate was associated with better survival [12]. Another study was designed to confirm the survival rates and prognostic factors in young children with nonmetastatic MB treated with postoperative chemotherapy alone. The results revealed that postoperative systemic multiagent chemotherapy and intraventricular MTX appears to be a promising treatment for young patients with desmoplastic/nodular MB or MB with extensive nodularity [1]. Even though, it remains unclear whether MTX intrathecal injection should be contraindicated for MB. Interestingly, a study showed that the avoidance of CRT is associated with good long-term neurocognitive outcomes for young children with ALL, while the dose of intravenous MTX did not affect these outcomes [13]. As such, it has been recommended to reconsider the use of intrathecal MTX in the treatment of MB. Indeed, intrathecal MTX was an independent prognostic factor for good prognosis in our study, even though there was no statistical significance. Multivariate Cox proportional hazard analysis revealed that good prognosis was associated with R0 resection, no diffuse anaplasia histology, and M0 status. However, the *p*-values of these factors showed no statistical significance, which might be attributed to the relatively small sample size of the two groups.

Moreover, to our knowledge, the highest reported 5-year PFS for metastatic MB was 71%, achieved with carboplatin at 35 mg/m^2^ for 30 doses before daily radiotherapy [14]. A 5-year PFS of 62–70% for children with high-risk disease has been reported after radiotherapy combined with various chemotherapeutic agents used during and after radiotherapy [11, 15–17]. Typically, a 70% 5-year PFS was reported in the St. Jude prospective study that adopted post-radiotherapy high-dose chemotherapy and stem cell rescue [16]. In our study, the 5-year PFS was 70.99%, and the 5-year OS was 72.99% for the MTX group. There were 32 patients in the MTX group, 28 of whom were included in molecular analyses. Except for the unknown molecular subtype of these 4 patients, none of the remaining cases belonged to the WNT subtype, suggesting that about 10% of all MB patients had excellent prognoses [18]. The 5-year PFS for the MTX group was 70.99%, which is an improvement compared to previously reported PFS rates, suggesting a benefit for patients who received intrathecal MTX combined with chemotherapy and radiotherapy. Moreover, our study reported a good 5-year OS rate and significant differences in the 5-year PFS and 5-year OS between MTX and control groups.

Interestingly, all patients with acute leukoencephalopathy were females with group 4 MB. Although the mechanism of MTX-related leukoencephalopathy remains unclear, several mechanisms have been proposed. For instance, certain gene polymorphisms such as single nucleotide polymorphisms in the methylenetetrahydrofolate reductase [19], glutathione S-transferase Pi 1genes [20], and ATP binding cassette subfamily B member 1 [21]. Furthermore, a recent study revealed that adenosine receptors and high cumulative doses of systemic MTX administration are significantly associated with MTX-related leukoencephalopathy in patients with hematological malignancies [22]. However, no study has focused on the mechanisms of MTA in inducing leukoencephalopathy in MB patients. Leukoencephalopathy observed during active treatment was referred to as “acute leukoencephalopathy” in our study [23]. The biological foundation of group 4 tumors is poorly understood, although it has been established that the key alternation is KDM6A, a histone demethylase. In a study on triple-negative breast cancer, recruitment of KDM6A was associated with tumor recurrence. Inhibition of adenosine receptors A2BR delayed tumor recurrence in vivo [24]. These findings suggest a potential relationship between the two genes. Further research is necessary to uncover the molecular mechanisms connecting KDM6A, adenosine receptors, and susceptibility to acute leukoencephalopathy in group 4 MB.

It has been established that 80% of children with cancer worldwide live in low-to-middle income countries and have inferior outcomes than children living in high income countries [25, 26]. It is widely thought that delays in diagnosis and treatment compromise survival [27]. Considering the excellent 5-year PFS, adding intrathecal MTX to high-risk MB patients of both SHH and group 3 subgroups is recommended as a primary option under certain circumstances.

Nevertheless, several limitations should be noted in our study. This was a retrospective study, and the sample size (especially for the control group) was relatively small. Besides, compared with the MTX group, patients in the control group were diagnosed earlier when surgical techniques were less advanced, making it challenging to directly compare the two groups’ outcomes. R + status was prevalent in the control group, and it is difficult to draw robust conclusions about the effect of incomplete resection on survival in this small cohort. Besides, long-term neurocognitive outcome data were limited to a small subset of patients.

## Electronic supplementary material

Below is the link to the electronic supplementary material.

Supplementary Material 1

## Data Availability

The patient data utilized in this study were accessed from the medical records room of the First Hospital of Jilin University. Since these datasets consist of files stored within our hospital’s medical records room, they are not available for public access. However, interested parties may request access to this information from the corresponding author through reasonable means.

## Abbreviations

ALL: Acute lymphoblastic leukemia
CBF: Cerebral blood flow
CNS: central nervous system
CSF: Cerebrospinal fluid
CI: Confidence interval
HR: Hazard ratios
IgG: Immunoglobulin G
IQ: Intelligence quotient
MB: Medulloblastoma
MRI: Magnetic resonance imaging
MRS: Magnetic resonance spectroscopy
MTX: Methotrexate
PFS: Progression-free survival
OCB: Oligoclonal band
OS: Overall survival
